# Le soutien familial du patient cancéreux: qu'en est-il du Maroc?

**DOI:** 10.11604/pamj.2013.15.64.2851

**Published:** 2013-06-21

**Authors:** Meryam Ben Ameur El Youbi, Saoussane Kharmoum, Hassan Errihani

**Affiliations:** 1 Service d'Oncologie Médicale, Institut National d'Oncologie, Rabat, Maroc

**Keywords:** Soutient moral, patient cancéreux, Maroc, moral support, cancer patient, Morocco

## Aux éditeurs du Journal Panafricain de Médecine

Le cancer est une affection grave et complexe de par ses multiples retentissements psychiques et sociaux aussi bien sur le patient lui-même que sur son entourage familial. L'annonce du diagnostic de cancer reste un événement à la fois inattendu, triste et inacceptable pour le patient et sa famille [1]. Une situation qui constitue une véritable “mise à l'épreuve”; pour la famille, mobilisant ainsi pour y faire face, toutes les ressources psychiques et même matérielles de chacun [2].

En cancérologie, le modèle de la famille marocaine est à la fois assez particulier et intéressant à examiner de près. En effet, un patient cancéreux marocain tout au long de son parcours avec la maladie, ne se présentera jamais seul dans la structure de soins, des fois même, toute la famille peut l'accompagner à l'hôpital. Nous nous sommes interrogés sur les raisons de cet afflux collectif et de cette solidarité que nous ne retrouvons pas dans les sociétés occidentales [3].

L'accompagnement du patient cancéreux dans son malheur est un moment opportun pour la famille marocaine de prouver son dévouement et son attachement vis-à-vis de cette personne malade. Pour elle, c'est aussi un devoir selon les normes culturelles, sociales et religieuses. Le patient cancéreux peut être une personne analphabète ou ignorante justifiant la présence d'une personne d'un certain niveau culturel afin de faciliter l'échange entre le patient et le médecin, ou il peut s'agir tout simplement d'une personne âgée ou très jeune, nécessitant une assistance par un ou plusieurs membres de la famille lors des différentes démarches au sein de la structure hospitalière. Accompagner le malade, c'est aussi un moment privilégié pour rencontrer le médecin traitant. Si c'est une annonce, la famille en profite généralement pour demander au médecin de ne pas prononcer le mot “cancer”; devant le patient et de le substituer par des termes moins tabous et alarmants, ou encore de “maquiller” le pronostic afin d'épargner le patient cancéreux de la souffrance psychique qui en découlera.

Le soutien apporté par la famille marocaine au patient cancéreux peut être certes bénéfique. Cependant, il peut présenter plusieurs aspects négatifs; tout d'abord priver le patient de son légitime droit de connaitre la vérité sur sa pathologie et le pronostic de cette dernière sous prétexte de le ménager psychiquement, le priver aussi du droit de choisir son plan de traitement si plusieurs options thérapeutiques lui sont proposées par son médecin. Par ailleurs, une consultation médicale en présence de plusieurs personnes peut être source de divergences dans les avis, de conflits familiaux, d'encombrement dans la salle de consultation et de difficultés de concentration pour le médecin, entravant ainsi le bon déroulement de la consultation médicale. A ce stade là, le rôle du médecin s'inscrit dans plusieurs niveaux: la discussion se fera avec un seul interlocuteur désigné par la famille elle-même, cette personne bien entendu servira d'intermédiaire entre le médecin et les autres membres de la famille, elle se chargera aussi d'assister à la consultation et ne devra en aucun moment décider pour le patient. Le médecin de son coté devra s'adresser au patient dans une langue claire et simple et l'intégrer continuellement dans la discussion et la décision thérapeutique.

Un autre exemple qui témoigne de la profondeur de cette solidarité est celui de la fin de vie. En effet, la majorité des familles marocaines insistent pour récupérer de l'hôpital leur parent en fin de vie ou agonisant, afin qu'il s'éteigne chez lui entouré par les siens, le laisser mourir à l'hôpital constitue une véritable honte dans la société marocaine. Contrairement aux pays occidentaux, où mourir avec dignité selon la famille du patient cancéreux ne conçoit que dans le cadre d'un accompagnement médicalisé de la fin de vie [4]. Dans ces sociétés, ceci est à l'origine d'une importante augmentation de la durée et des frais d'hospitalisation des patients cancéreux.

## Conclusion

Le modèle de la solidarité des familles marocaines en cancérologie diffère totalement de celui des familles occidentales. Certes, la société marocaine ne dispose pas des mêmes moyens et ressources dans le domaine de cancérologie que dans les pays développés, mais elle a réussi à garder cet aspect singulier et gratifiant de soutien et de solidarité vis-à-vis du parent cancéreux, puisés sans doute au fin fond de ses nobles valeurs culturelles, sociales et religieuses.
